# PHGDH: a novel therapeutic target in cancer

**DOI:** 10.1038/s12276-024-01268-1

**Published:** 2024-07-01

**Authors:** Chae Min Lee, Yeseong Hwang, Minki Kim, Ye-Chan Park, Hyeonhui Kim, Sungsoon Fang

**Affiliations:** 1 Graduate School of Medical Science, Brain Korea 21 Project, Yonsei University College of Medicine, Seoul, Republic of Korea; 2 Department of Biomedical Sciences, Gangnam Severance Hospital, Yonsei University College of Medicine, Seoul, Republic of Korea; 3 Chronic Intractable Disease for Systems Medicine Research Center, Yonsei University College of Medicine, Seoul, Republic of Korea; 4 Severance Institute for Vascular and Metabolic Research, Yonsei University College of Medicine, Seoul, Republic of Korea

**Keywords:** Cancer metabolism, Cancer microenvironment

## Abstract

Serine is a key contributor to the generation of one-carbon units for DNA synthesis during cellular proliferation. In addition, it plays a crucial role in the production of antioxidants that prevent abnormal proliferation and stress in cancer cells. In recent studies, the relationship between cancer metabolism and the serine biosynthesis pathway has been highlighted. In this context, 3-phosphoglycerate dehydrogenase (PHGDH) is notable as a key enzyme that functions as the primary rate-limiting enzyme in the serine biosynthesis pathway, facilitating the conversion of 3-phosphoglycerate to 3-phosphohydroxypyruvate. Elevated PHGDH activity in diverse cancer cells is mediated through genetic amplification, posttranslational modification, increased transcription, and allosteric regulation. Ultimately, these characteristics allow PHGDH to not only influence the growth and progression of cancer but also play an important role in metastasis and drug resistance. Consequently, PHGDH has emerged as a crucial focal point in cancer research. In this review, the structural aspects of PHGDH and its involvement in one-carbon metabolism are investigated, and PHGDH is proposed as a potential therapeutic target in diverse cancers. By elucidating how PHGDH expression promotes cancer growth, the goal of this review is to provide insight into innovative treatment strategies. This paper aims to reveal how PHGDH inhibitors can overcome resistance mechanisms, contributing to the development of effective cancer treatments.

## PHGDH: targeting one-carbon metabolism for cancer therapy

Serine is important in DNA copying and cancer cell growth as it helps produce antioxidants and other substances. This detailed review explores the role of 3-phosphoglycerate dehydrogenase, an enzyme, in cancer, particularly its role in serine creation and its potential as a treatment target. The review combines results from different studies, using various experimental methods to understand how PHGDH affects cancer cell behavior and treatment responses. Researchers suggest that targeting PHGDH could be a promising strategy for cancer treatment, potentially improving outcomes for patients with tumors that overproduce PHGDH. The authors call for more research to fully understand PHGDH’s role in cancer and to develop effective inhibitors that could be used in clinical settings. This work advances our understanding of cancer metabolism and opens new possibilities for treatment.

This summary was initially drafted using artificial intelligence, then revised and fact-checked by the author.

## Introduction

Serine provides one-carbon (1C) units for de novo purine and deoxythymidine synthesis, which are crucial for DNA replication during cellular proliferation^1^. Serine plays a role in the production of glutathione, hypotaurine, and NADPH, which are antioxidants that can prevent the abnormal proliferation of cancer cells and stresses associated with these cells^2,3^.

3-Phosphoglycerate dehydrogenase (PHGDH) is known to play important roles in cancer progression and migration^4,5^. PHGDH has the capacity to stabilize the oncogenic forkhead box protein M1, a factor implicated in tumor invasion, initiation, and proliferation processes^6^. Increased PHGDH activity in various cancer cells is mediated through genetic amplification, posttranslational modification, enhanced transcription, and allosteric regulation^7,8^. Therefore, PHGDH is now recognized as a significant factor in cancer research.

PHGDH is one of three sequential enzymes involved in the serine synthesis pathway (SSP). The SSP is an important component of glycolysis and contains the sequential enzymes phosphoserine aminotransferase 1 and phosphoserine phosphatase, in addition to PHGDH^9,10^. The activity of this pathway is affected by various processes considered important in cancer research, such as antioxidant production and methylation reactions. PHGDH is known to be a substantial contributor to the importance of this pathway in cancer growth because PHGDH plays the most important role in serine production^11^. Research on the proteins that regulate PHGDH is ongoing, and it can be seen that PHGDH does not act alone in cancer but has factors through which partnerships are formed^7,12,13^.

In this review, we thoroughly examine the pivotal role of PHGDH and its implications for cancer treatment. With insights drawn from an in-depth analysis of existing research on metabolic processes, our primary focus is on elucidating the importance of PHGDH across various cancers to propose its potential as a promising therapeutic target. The objective is to contribute to an improved understanding of innovative treatment approaches for a spectrum of cancers.

## PHGDH isoforms

PHGDH is expressed in all organisms, with its basic structural forms categorized into types I, II, and III. Type I PHGDH proteins are distinguished by their extensive structure, with one nucleotide-binding domain sandwiched between two substrate-binding domains. In addition, these proteins contain an allosteric substrate-binding (ASB) domain and an aspartate kinase-chorismate mutase–tyrosinase A prephenate dehydrogenase (ACT) domain. The unique ASB domain, which acts as a substrate binding control site, sets Type I PHGDH proteins apart from the other structural forms. Immediately downstream of this domain is the ACT domain, which is also present in Type II PHGDH proteins. In certain species, the ACT domain is known to be a serine binding site that functions as a feedback inhibitor. Type I PHGDH proteins with these characteristics can be found in mammals, plants, and bacteria.

Type II PHGDH proteins, a form of Type I PHGDH proteins without an ASB domain, consist of one nucleotide-binding domain sandwiched between two substrate-binding domains and an ACT domain. This structural type is found in eukaryotes and bacteria.

**Fig. 1:**
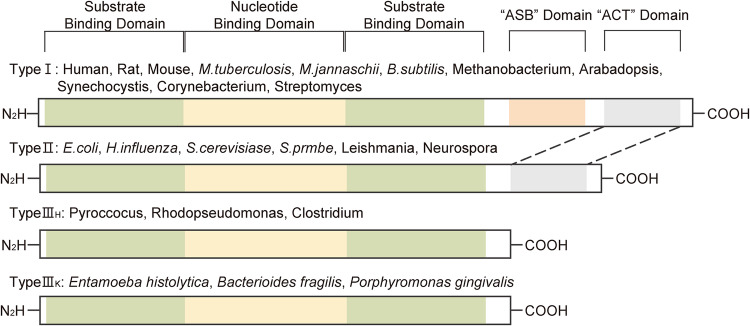
Isoforms of PHGDH in various organisms. Type I is dominant in humans, rodents, fungi, plant, and bacteria, while Types II and III are primarily found in bacteria. All four types share common features, such as a substrate-binding domain, nucleotide-binding domain, and amino and carboxyl termini. Notably, Type I PHGDH proteins possess an additional ASB domain and ACT domain, while Type II PHGDH proteins contain an ASB domain. Type III PHGDH proteins can be subdivided into two forms, which are determined by the presence of a histidine (type H) or a lysine (type K) in the active site. Allosteric substrate-binding (ASB); Aspartate kinase-chorismate mutase–tyrosinase A prephenate dehydrogenase (ACT).

Type III PHGDH proteins are characterized by the absence of both the ASB and ACT domains, making them the shortest among the three types. These proteins are composed of only two substrate-binding domains and one nucleotide-binding domain. Two polypeptide chain segments anchor the structure at the active cleft site. Type III PHGDH proteins are further divided into Type III_H_ and Type III_K_. If a lysine is present in the active site, the protein adopts the Type III_K_ structure, and if a histidine is present, it adopts the type III_H_ structure. Unlike other types, Type III PHGDH proteins do not exhibit a distinct organism-specific expression pattern. Type III_H_ can be found in Pyro coccus, Rhodopseudomonas, and Clostridium, while Type III_K_ has been identified in *Entamoeba histolytica*, *Bacteroides fragilis*, and *Porphyromonas gingivalis*^14,15^ (Fig. 1).

### Amino acid residues targeted for modifications in PHGDH

**Fig. 2:**
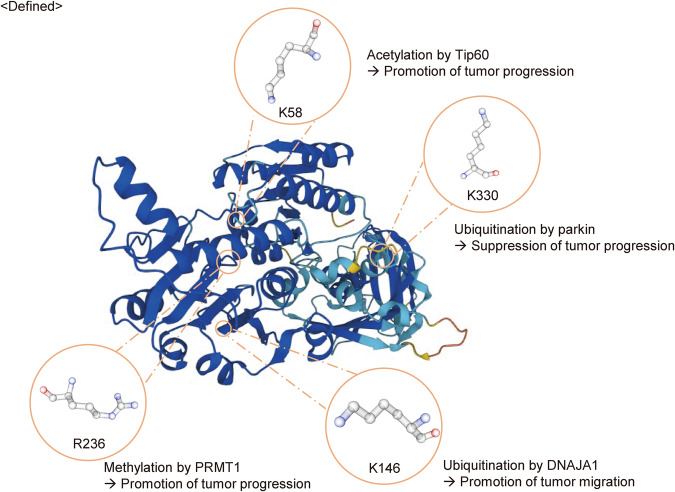
Posttranslational modifications of amino acid residues in PHGDH. Lysine (K) 58, K146, K330, and arginine (R) 236 play a role in regulating the physiological functions of PHGDH. The structural image was sourced from the AlphaFold Protein Structure Database (https://alphafold.ebi.ac.uk/).

Modifications of the amino acid residues of PHGDH are crucial for its ability to control tumor progression. Essential amino acid residues, including lysine (K) 58, K146, K330, and arginine (R) 236, have been identified in monoubiquitination, acetylation, and methylation, regulating the physiological function of PHGDH (Fig. 2).

#### K58

The acetylation of lysine K58, the primary acetylation site on PHGDH, stimulates cancer cell proliferation. One study showed that the E53 ubiquitin ligase ring finger protein 5 is essential for PHGDH protein degradation in breast cancer. The degradation of PHGDH by ring finger protein 5 inhibited breast cancer cell growth, and the acetylation of PHGDH at K58 disrupted the interaction between ring finger protein 5 and PHGDH and promoted breast cancer cell proliferation. K58 is acetylated by Tip60, and this event promotes cancer progression. Tip60 is an acetyltransferase of PHGDH and increases the K58 acetylation of PHGDH when overexpressed^13,16^.

#### K146

Monoubiquitination of K146 promotes cancer migration, and K146 is ubiquitinated by DnaJ homolog subfamily A member 1. Monoubiquitination of PHGDH at K146 increases its interaction with the molecular chaperone DnaJ homolog subfamily A member 1, facilitating PHGDH tetramerization and augmenting PHGDH activity. This ultimately increases the level of S-adenosyl methionine in cells. The heightened abundance of S-adenosyl methionine results in activation of the methyltransferase SET domain containing 1A. This activation impacts the promoters of laminin subunit gamma 2 and cysteine-rich angiogenic inducer 61, fostering metastasis in colorectal cancer cells^16,17^.

#### K330

Parkin induces ubiquitination at K330 of PHGDH. Mutation of this residue from lysine to arginine (K330R) resulted in diminished Myc-Parkin-mediated ubiquitination of PHGDH. This mutation not only decreased the ability of Parkin to reduce the PHGDH protein level in cells but also extended the decay time of the protein. The expression of Myc-Parkin failed to decrease the intracellular level of the K330R PHGDH protein, indicating that K330 in PHGDH is the primary ubiquitination site targeted by Parkin^18,19^.

#### R236

The serine level is increased in hepatocellular carcinoma (HCC). However, there is also a decrease in the expression of PHGDH, which is the key enzyme controlling the rate of serine production. Importantly, the serine level is increased because the PHGDH protein is methylated at arginine 236 (R236) by protein arginine methyltransferase 1, which stimulates the catalytic activity of PHGDH. Therefore, protein arginine methyltransferase 1-mediated PHGDH methylation increases synthesis of serine and alleviation of oxidative stress, thereby promoting the growth of HCC^7^.

### Modulators of PHGDH enzymatic activity

#### p300

p300 is a transcriptional coactivator known to modify the chromatin environment and link transcription factors to the primary transcription machinery^20^. In colon cancer cells, ATF3 interacts with p300, and p300 increases transcriptional activity by recruitment to serine synthesis-related proteins such as PHGDH. More specifically, p300 binds to the PHGDH/phosphoserine aminotransferase 1 enhancer/promoter region where ATF3 is bound. This binding is further promoted in the absence of serine. Notably, in ATF3 knockout cells, the amount of p300 attached to the enhancer site of PHGDH was significantly reduced. Therefore, ATF3 is crucial for p300 recruitment to SSP genes under serine-deficient conditions^21^.

#### Josephin-2

Several studies have indicated that deubiquitinating enzymes have potential use for HCC treatment^22–24^. A deubiquitinating enzyme, Josephin-2 promotes the cancerous advancement of HCC cells both in vitro and in vivo by stabilizing the PHGDH protein. Suppression of the malignant phenotype in HCC cells through Josephin-2 knockdown is attenuated by PHGDH overexpression, underscoring the role of PHGDH in mediating the role of Josephin-2 in promoting HCC progression. In summary, Josephin-2 has emerged as an oncogene in HCC, and the Josephin-2/PHGDH pathway plays a crucial role in HCC progression^25^.

#### Cofilin1

Elevated expression of Cofilin1 (CFL1) is observed in HCC patients^26^, particularly in those who are resistant to sorafenib, and is associated with an unfavorable prognosis. CFL1 promotes the depolymerization of F-actin to G-actin. This impairs the interaction between the redox-sensitive repressor Kelch-like ECH-associated protein 1 and the ubiquitous transcription factor erythroid 2-related factor 2 (Nrf2), thus increasing the nuclear translocation of Nrf2. This ultimately results in increased PHGDH transcription and the promotion of serine synthesis and metabolism. Suppression of CFL1 expression in various HCC cell lines results in decreased PHGDH mRNA and protein levels, suggesting that CFL1 influences the expression of PHGDH through transcriptional mechanisms. The positive correlation between CFL1 and PHGDH expression was found to be particularly pronounced in the tumor tissue of sorafenib nonresponders in a population of HCC patients. In brief, increased CFL1 expression hinders the Kelch-like ECH-associated protein 1–Nrf2 interaction, leading to increased PHGDH transcription and Nrf2 nuclear translocation^27^.

## Physiological roles of PHGDH in one-carbon metabolism

**Fig. 3:**
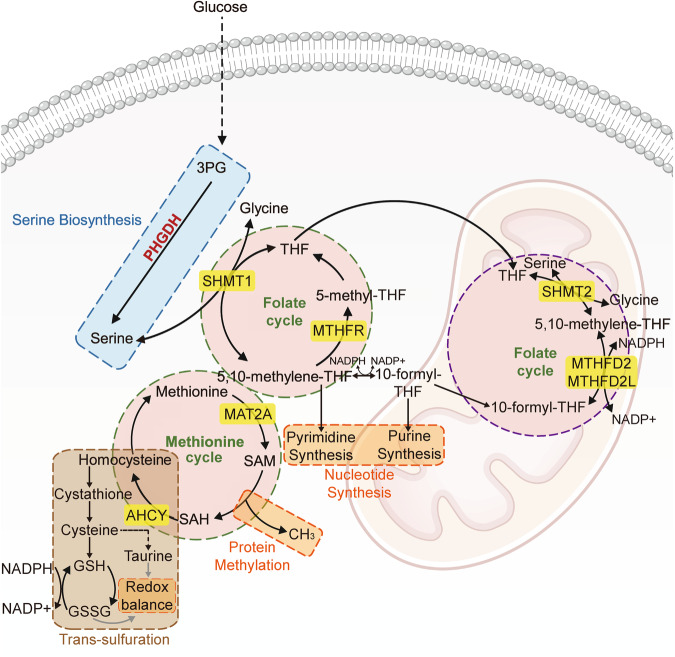
Overview of 1C metabolism. PHGDH functions as the rate-limiting enzyme in serine synthesis, governing 1C metabolism. In 1C metabolism, PHGDH has the capacity to modulate the redox balance, nucleotide synthesis, and epigenetic processes. The folate cycle and the methionine cycle are the two primary cyclic processes in 1C metabolism. 3-phosphoglycerate (3PG); Tetrahydrofolate (THF); Methylenetetrahydrofolate reductase (MTHFR); Methionine adenosyltransferase 2 A (MAT2A); S-adenosyl methionine (SAM); S-adenosylhomocysteine (SAH); adenosylhomocysteinase (AHCY); Glutathione (GSH); Glutathione disulfide (GSSG); Serine hydroxymethyltransferase 1 (SHMT1); Serine hydroxymethyltransferase 2 (SHMT2); Methylenetetrahydrofolate dehydrogenase 2 (MTHFD2); Methylene tetrahydrofolate 2-like (MTHFD2L).

The essence of 1C metabolism lies in the intricate interplay between the folic acid and methionine cycles. The reactions in these interconnected cycles utilize the 1C units derived from glycine and serine, which function as essential raw materials to integrate cellular metabolism^28^. The metabolites encompass a diverse range of products, including glutathione, nucleotides, and S-adenosyl methionine. These substances play crucial roles in the synthesis of DNA and RNA, and they are vital for maintaining cellular functions overall^29^ (Fig. 3).

### Folate cycle

Folate metabolism plays a crucial role in activating and supplying 1C units for various biosynthetic processes^30^. Folic acid serves as a transporter for 1C units, and its covalent modification involves polyglutamylation through interaction with vitamins^31^. In this intricate process, the synthesis of purines from glycine and serine serves as a fuel source for mitochondrial enzymes^1^. This process is initiated by the action of serine hydroxymethyl transferase 1, which converts serine to glycine. The generated 1C unit influences tetrahydrofolate (THF), facilitating the bidirectional transformation of serine into glycine, facilitated by serine hydroxylmethyltransferase-1 or serine hydroxylmethyltransferase-2. Ultimately, this process yields 5,10-methylene-THF in both the cytoplasm and mitochondria^32^. 5,10-methylene-THF impacts two nucleotide synthesis processes in the cytoplasm^33^. Specifically, it plays a direct role in pyrimidine synthesis and, upon its conversion to 10-formyl-THF, provides methyl groups for purine synthesis. In the second reaction, the oxidation of NADPH to NADP^+^ occurs^34^. Alternatively, 5,10-methylene-THF is reduced to 5-methyl-THF by methylene-THF reductase^35^. In the folate cycle, 5-methyl-THF is crucial for contributing to the generation of THF through 1C pathways. In mitochondria, the conversion of 5,10-methylene-THF to 10-formyl-THF is facilitated by either methylene-THF dehydrogenase 2 or methylene-THF dehydrogenase 2-like. This process requires the reduction of NAD to NADH^32^.

### Methionine cycle

In conjunction with the folate cycle, the methionine cycle constitutes another crucial facet of 1C metabolism^36^. The methionine cycle commences with the conversion of methionine to S-adenosyl methionine facilitated by methionine adenosyltransferase 2A. Of importance here is the association with protein methylation, which originates from the synthesis of S-adenosyl methionine. Histone methyltransferases assist in transferring a methyl group from S-adenosyl methionine to a histone substrate^37^. S-adenosyl methionine serves as the primary supplier of methyl groups within the cell and is utilized by methyltransferases, ultimately leading to its conversion into S-adenosyl homocysteine. S-adenosyl methionine homocysteine is transformed into homocysteine by adenosylhomocysteinase. Then, homocysteine can enter into two pathways: one in which it is recycled back to methionine and another in which it is converted to cysteine via the trans-sulfuration pathway. In the former case, methionine synthase can reverse this compound's transformation, thereby closing the methionine cycle^38–40^. In the latter case, homocysteine is converted to cysteine, which is then utilized to maintain the redox balance by contributing to the synthesis of glutathione and taurine. Glutathione serves as a reversible antioxidant buffer capable of removing reactive oxygen species (ROS), after which it is oxidized reversibly to glutathione disulfide^41–44^.

## Physiological roles of PHGDH in cancers

An increase in PHGDH expression triggers an increase in de novo serine biosynthesis, which is intricately linked to cell growth and proliferation^45^. Elevated levels of serine have diverse impacts on different cellular processes, contributing to glycine synthesis, aiding as part of homocysteine to cysteine in the methionine cycle, and supporting protein synthesis^46^. In addition, serine integration into lipids contributes to phosphoserine production. These interconnected processes promote cellular biomass production and nucleotide synthesis, which are crucial for the rapid growth of cancer cells^47^. Numerous studies have consistently revealed significant upregulation of PHGDH expression across various cancer types, in which it is correlated with tumor aggressiveness^48–53^. Recent studies have provided several references for further understanding the oncogenic potential of PHGDH and suggesting therapeutic possibilities for PHGDH inhibition.

### Lung cancer

Lung cancer exhibits abnormal serine metabolism with increased SSP activity, which influences malignant progression and therapeutic resistance^48^. PHGDH stands out as the key player in this pathway, demonstrating heightened activity^54^.

In non-small cell lung cancer (NSCLC), PHGDH expression is greater in tumor tissues than in matched adjacent lung tissues^7^. Furthermore, elevated PHGDH mRNA levels in human NSCLC tissues relative to neighboring normal tissues were identified using one-step qRT‒PCR. PHGDH expression also demonstrated a positive correlation with the TNM (tumor status, lymph node metastasis, distant metastasis) stage, as revealed by immunohistochemical analysis. This increase aligns with evidence demonstrating that patients with a high PHGDH level have a shorter survival time than those with a low PHGDH level, suggesting the possible involvement of PHGDH in cancer progression in NSCLC patients^55^.

Elevated PHGDH expression is evident in lung adenocarcinoma, a property associated with an unfavorable prognosis. Cell culture models with elevated PHGDH expression display increased proliferation and migration, and these results establish a connection to glutathione and pyrimidines through serine metabolism^56^.

### Colorectal cancer

As the tumorigenic role of PHGDH is being studied in patients with colorectal cancer (CRC), the therapeutic potential of PHGDH is being consistently suggested. Recently, hypoxic conditions were shown to increase PHGDH expression in human CRC cell lines. PHGDH inhibition was shown to increase ROS levels, leading to increased radiosensitivity^57^. Another study showed that additional treatment of CRC cell lines cultured under serine- and glycine-deficient conditions resulted in a notable decrease in SSP enzyme expression. Similarly, combination treatments with drugs and inhibitors significantly inhibited patient-derived organoid growth. This finding underscores the concept that the response triggered by serine and glycine starvation and PHGDH deletion is ultimately a response induced by the loss of PHGDH activity^11^. In CRC patient tissues, immunohistochemical analysis has consistently shown that PHGDH displays heightened expression levels that positively correlate with both TNM stage and tumor size. Furthermore, within the CRC patient group, higher PHGDH expression was associated with a worse prognosis^58^. These findings collectively suggest that elevated PHGDH expression may play an important role in the progression and metastasis of CRC in patients.

### Pancreatic cancer

Pancreatic cancer cell lines exhibit substantial diversion of glycolytic metabolism toward increased serine and glycine synthesis flux^51^. Moreover, the inhibition of PHGDH in cancer cells suppresses cell proliferation and tumorigenesis. In a xenograft mouse model of pancreatic cancer, tumor-bearing mice showed significant extension of survival compared to that of mice in the control group. Moreover, analysis of mRNA expression indicated that lower PHGDH mRNA expression was associated with a more favorable prognosis than that observed in the control group. Suppressing PHGDH led to a reduction in the number of colonies and significantly disrupted intercellular tight junctions within individual colonies in the colony formation assays. Another study demonstrated that the growth and ability to form colonies of pancreatic cancer cells were significantly decreased upon PHGDH knockdown^59^.

Similarly, in human pancreatic cancer tissues, the proportion of cells with positive PHGDH expression was greater than that in adjacent nontumor tissues. Furthermore, elevated PHGDH levels correlated significantly with larger tumor size, lymph node metastasis, and more advanced TNM stage. Concurrently, patients exhibiting high PHGDH expression also demonstrated reduced overall survival and disease-free survival rates than those with low PHGDH expression^59^. These consistent findings emphasize that PHGDH can be utilized as a diagnostic marker and is highly important as a marker for the early detection of pancreatic cancer.

Under serine-deficient conditions, p53 expression is strongly induced. PHGDH expression in pancreatic ductal adenocarcinoma under conditions of serine depletion exhibits an increase consistent with the cellular growth pattern. This finding underscores the implication of inducing PHGDH expression under conditions of serine deficiency to maintain the intracellular serine level and promote the growth of pancreatic ductal adenocarcinoma cells. Moreover, pancreatic ductal adenocarcinoma cells subjected to PHGDH siRNA transfection displayed limited growth under standard conditions, whereas the growth of S2-VP10 cells subjected to PHGDH siRNA transfection was substantially diminished in the absence of serine. This highlights the considerable influence of PHGDH on the growth of pancreatic ductal adenocarcinoma under conditions of serine deficiency^60^.

### Breast cancer

In breast tumorigenesis in vivo, PHGDH is associated with frequent copy number increases and exhibits elevated expression in 70% of estrogen receptor-negative breast cancers. Breast cancer cells with elevated PHGDH expression also exhibit heightened serine synthesis, and inhibiting PHGDH significantly reduces cell proliferation and inhibits serine synthesis. Hence, the overexpression of PHGDH in specific breast cancer types leads to increased reliance on the serine pathway^61^.

In various breast cancer cell lines, PHGDH expression was found to be highly dependent on hypoxia-inducible factor expression in a hypoxic environment. This effect was notably observed in various types of breast cancer cells: estrogen receptor-positive cells (ZR75.1); estrogen and progesterone receptor-positive cells (MCF-7); estrogen, progesterone, and HER2 receptor-positive cells (HCC-1954); and triple-negative breast cancer (TNBC) cells lacking any of these receptors (MDA-MB-231, SUM-149). Notably, PHGDH knockdown had no impact on the proliferation of estrogen and progesterone receptor-positive breast cancer cells. However, it increased the proliferation of TNBC cells. This finding suggested that inactivation of PHGDH has the most dramatic effects under hypoxic conditions, leading to increased cell death^62^.

### The role of PHGDH in metastasis

Tumor metastasis is responsible for 90% of cancer-related deaths and marks the final stage in the adaptive evolution of cancer cells as they navigate different tissue environments^63,64^. PHGDH not only influences the various cancer types mentioned earlier but also plays a crucial role in tumor metastasis.

PHGDH is a highly expressed oncogene in lung cancer due to the active serine synthesis in this disease, and its elevated expression is positively associated with lymph node metastasis in NSCLC^55^. This observation demonstrated that proliferation and migration were accelerated in cells overexpressing PHGDH.

In CRC-adjacent tissues, high PHGDH expression was found to be correlated with advanced TNM stage and a large tumor size^58^. In addition, the expression level of PHGDH was higher in the tissues of patients with metastatic recurrence of CRC than in those of patients without metastatic recurrence of CRC. It was confirmed that PHGDH expression was increased in the livers of patients who had metastasis to the liver. Moreover, depleting PHGDH significantly suppressed tumor metastasis. This shows that the level of PHGDH activity has a significant impact on tumor cell migration and CRC metastasis^17^.

It was reported that PHGDH expression and metastasis are closely associated in breast cancer. Within primary breast tumors, intratumor PHGDH expression is evident, and a low level of PHGDH expression is a marker of metastasis in patients. In mice, circulating tumor cells and early metastatic lesions were found to exhibit low expression of the PHGDH protein. This phenomenon ultimately facilitated increased cell dissemination and the formation of metastases^65^. In addition, PHGDH expression was found to be increased in a model of aggressive TNBC brain metastasis. In TNBC brain metastasis-bearing mice injected with PHGDH short hairpin RNA, brain metastasis was significantly reduced, and overall survival was improved. In addition, patients with brain metastasis of breast cancer were found to have higher PHGDH expression than did patients with extracranial metastases, including lung, liver, and ovarian metastases, of breast cancer. This finding implies that PHGDH plays a pivotal role in the metastasis of breast cancer^66^. In vivo, in lung metastases derived from breast cancer, rapamycin had an antitumor effect on reducing the tumor area only in PHGDH-overexpressing breast cancer group. This is noteworthy because the heightened presence of PHGDH in lung metastases of breast cancer is essential for increasing sensitivity to mTORC1 signaling and the subsequent positive response to rapamycin, but this dependency is not observed in primary tumors^67^.

The degree of PHGDH activation by ZEB1 is important in lung metastasis of HCC. ZEB1 significantly contributes to the transcriptional activation of PHGDH, influencing SSP flux during the development and progression of HCC. Therefore, the ZEB1-PHGDH regulatory axis affects the migration, invasion, and tumorigenicity of HCC cells. Restoration of function in functionally compromised HCC cells can be achieved through the re-expression of PHGDH. Consequently, increased levels of ZEB1 and PHGDH are critical factors in both HCC tumor formation and the processes of migration and invasion^68^.

One study showed that PHGDH expression and metastasis are positively correlated in cervical lymph node metastasis of papillary thyroid cancer. PHGDH regulates the stemness of cancer cells and induces thyroid cancer cell proliferation and tumorigenesis, and is also associated with thyroid cancer aggressiveness^69^.

A correlation between PHGDH expression and metastasis stage has been reported in patients with esophageal squamous cell carcinoma. The findings revealed a significant association between PHGDH expression and the pathological stage of esophageal squamous cell carcinoma. In addition, the T and M stages within the TNM classification demonstrate a substantial relationship with the prognosis of esophageal squamous cell carcinoma patients^70^.

In endometrial cancer, the knockdown of PHGDH was found to significantly inhibit the migration of cancer cells in vitro. This inhibition of PHGDH expression not only reduced the proliferation of endometrial cancer cells but also induced apoptosis, leading to a decrease in the metastasis of these cancer cells^71^.

### The role of PHGDH in anticancer drug resistance

Cancer cells adapt their metabolic processes, utilizing compensatory metabolic pathways, to survive treatments for different types of cancer. This impaired sensitivity of tumors to anticancer agents ultimately results in partial treatment responses and the development of resistance over time^72^. The expression of PHGDH also exerts another effect by inducing resistance to treatment. The extent of increased PHGDH expression depends on specific metabolic pathways. Because these specific metabolic pathways are direct targets of cancer therapies, inhibitors targeting PHGDH in tumors with expression of components of these pathways may lead to the development of resistance.

Sunitinib, a multikinase inhibitor used to treat renal cell carcinoma (RCC), interacts with hypoxia-inducible factor (HIF) to inhibit its activity. HIF is crucial for RCC progression and the development of resistance to antiangiogenic multikinase and mTOR inhibitors. While HIF2α antagonists are utilized for RCC treatment, concerns about resistance remain. Significantly, the signaling activated by HIF2α deficiency was identified as a mediator of sunitinib resistance and was accompanied by PHGDH upregulation and activation of the serine synthesis pathway. Treating RCC with a PHGDH inhibitor induces apoptosis and reduces the growth of HIF2α-deficient tumor cells. This finding unveiled serine biosynthesis as a potential therapeutic target for overcoming resistance to HIF2α antagonists mediated via PHGDH inhibition in advanced and metastatic clear cell RCC^73^.

Sorafenib is a standard treatment agent for HCC, but its use commonly leads to resistance. HCC cells activate PHGDH and the SSP to generate antioxidants and α-ketoglutarate, enabling them to survive the oxidative stress induced by sorafenib. Consequently, sorafenib activates the SSP by increasing PHGDH activation. If PHGDH is not activated, the levels of ROS increase, and sorafenib treatment induces apoptosis in HCC cells. Moreover, NRF2 and ATF4 can upregulate PHGDH. This upregulation leads to an increase in NADPH synthesis and the α-ketoglutarate level under conditions of sorafenib resistance. This process helps regulate redox homeostasis and contributes to resistance^74^.

In lung adenocarcinoma, PHGDH contributes to resistance to the therapeutic agent erlotinib. PHGDH controls the expression of transcripts related to DNA damage repair and nucleotide metabolism in NSCLC cells, contributing to the acquisition of erlotinib resistance. Through these processes, PHGDH ultimately increases the concentration of erlotinib in NSCLC cells, confirming the ability of PHGDH to regulate the proliferation and metabolic adaptation of these cells^75^.

Vemurafenib, a MAP kinase pathway inhibitor, is employed for the treatment of unresectable or metastatic melanoma harboring the *BRAF* V600E mutation. Specifically, by acting on the *BRAF* V600E protein, vemurafenib promotes cell migration and proliferation through ERK1/2. While *BRAF* inhibitors initially induce tumor regression, disease recurrence eventually develops due to acquired tumor resistance. Notably, PHGDH and other serine biosynthesis pathway proteins exhibit differential expression between vemurafenib-resistant and vemurafenib-sensitive cells. Resistant cells exposed to vemurafenib show higher or unchanged levels of all enzymes in the pathway, with an increased PHGDH level, after treatment. Depleting PHGDH with siRNA reduces the viability of resistant cells and induces cell death after vemurafenib treatment^76^.

Bortezomib, a proteasome inhibitor, is utilized for treating multiple myeloma. However, the emergence of widespread resistance has necessitated the identification of therapeutic targets for improved treatment outcomes. Resistance to bortezomib has been attributed to changes in glucose metabolism, and PHGDH expression has been correlated with bortezomib resistance in multiple myeloma. Continuous maintenance of serine deficiency was found to increase the cytotoxicity of bortezomib, and a significant increase in PHGDH expression was observed in CD138+ cells from patients with bortezomib-refractory multiple myeloma^77^.

Finally, the chemotherapeutic agents targeting PHGDH are cisplatin, doxorubicin, and 5-Fluorouracil (5-FU). Studies have also shown that PHGDH expression is increased in platinum-resistant ovarian cancer cells and tissues. The overexpression of PHGDH increased the survival rate of ovarian cancer cells upon exposure to cisplatin and increased their invasiveness and spheroid formation ability. In other words, increased PHGDH expression confers resistance to cisplatin on ovarian cancer cells^56,78^.

Doxorubicin is used as a chemotherapeutic agent for TNBC. However, its use is limited by dose-dependent toxicity and drug resistance, highlighting the need for novel treatment strategies. Doxorubicin induces damage to nucleotides, proteins, and lipids through the production of ROS, resulting in cell death. Therefore, the removal of ROS produced in response to doxorubicin is crucial for the survival of cancer cells. In this context, it has been discovered that the production of glutathione by PHGDH plays a protective role against doxorubicin-induced oxidative stress in TNBC cells. This finding implies that simultaneous treatment with doxorubicin and inhibition of PHGDH could be a more effective therapeutic approach than either strategy alone^79^.

5-FU plays a crucial role in colon cancer chemotherapy. While 5-FU is effective in elevating intracellular folate cofactor levels and inducing DNA damage, the challenge of drug resistance remains. Notably, an increase in the serine level was observed in 5-FU-resistant CRC cells, prompting the exploration of various regulatory mechanisms influencing PHGDH. These mechanisms included gene amplification, protein degradation, and transcriptional regulation. In addition, a study revealed that the p53 status and corresponding murine double minute-2 expression level are pivotal factors in the heightened serine demand during the development of 5-FU resistance in CRC cells^80^.

**Fig. 4:**
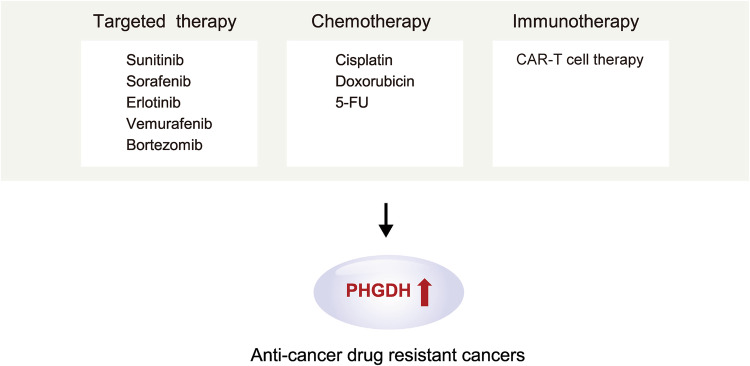
PHGDH is a novel anticancer therapeutic target for drug-resistant cancers. PHGDH is crucial for combating cancer drug resistance. 5-Fluorouracil (5-FU); Chimeric antigen receptor-T cell (CAR-T cell).

In one study, it was found that PHGDH-mediated endothelial cell metabolism contributes to glioblastoma resistance to chimeric antigen receptor-T-cell immunotherapy. This resistance was linked to the establishment of a vascular microenvironment and hypoxia, which negatively impacted immunity. This study confirmed that changes in the PHGDH expression level and serine metabolism were observed predominantly in tumor endothelial cells. Notably, signals from the tumor microenvironment induce PHGDH expression in endothelial cells, activating a redox-dependent mechanism that leads to hyperproliferation of endothelial cells. Consequently, deletion of the PHGDH gene from endothelial cells inhibits the excessive growth of blood vessels, eradicates hypoxia within the tumor, and promotes the infiltration of T cells into the tumor. Thus, inhibiting PHGDH activation enhances Tcell-mediated antitumor immunity, increasing the responsiveness of glioblastoma to chimeric antigen receptor-Tcell therapy^81^ (Fig. 4).

## PHGDH as a novel therapeutic target for cancer

The roles of PHGDH in various cancer types point toward promising options for novel treatment strategies. In a recent study, the combined application of the pyruvate kinase M2 inhibitor PKM2-IN-1 and the PHGDH inhibitor NCT-503 was investigated in human NSCLC A549 cells. This study included both in vitro and in vivo experiments, revealing increased levels of 3-phosphoglycerate and PHGDH. This elevated PHGDH was verified to result in inhibition of cell proliferation, leading to cell cycle arrest and eventual apoptosis. In simpler terms, the synergy between the two inhibitors leveraging the relationship between pyruvate kinase M2 and serine synthesis in NSCLC demonstrated a potent anticancer effect, effectively suppressing the growth of lung tumors^82^.

In CRC cells treated with the PHGDH inhibitor NCT-503, liver metastasis, and primary tumor growth were suppressed, and NCT-503 treatment reduced tumor cell migration, indicating the important role of PHGDH in tumor metastasis^17^. Furthermore, the use of NCT-503 led to a decrease in cellular respiration, along with reductions in ATP production and the maximum respiratory capacity in cells. Radiotherapy is a notable widely employed CRC treatment strategy. The results of mouse experiments confirmed the heightened effectiveness of combining NCT-503 treatment with radiotherapy.

Cisplatin and paclitaxel are commonly used for advanced pancreatic cancer treatment and exhibit inhibitory effects on PANC-1 cell proliferation. The combination of CBR-5884 treatment with PHGDH inhibition enhanced cisplatin- or paclitaxel-induced cytotoxicity in PANC-1 cells. In addition, triple combinations exhibited maximum cytotoxicity and aused a nearly 90% decrease in the viability of PANC-1 cells, indicating that PHGDH is a potential therapeutic target for increasing the efficacy of chemotherapy^51^.

In TNBC cells, the upregulation of PHGDH increases the flux of glucose into serine synthesis. Consequently, serine undergoes conversion to support glutathione synthesis to counteract the formation of ROS induced by doxorubicin. PHGDH inhibition through short hairpin RNA was found to induce oxidative stress, leading to increased sensitivity to doxorubicin^79^. In addition, PHGDH may be a target for reversing recurrence and resistance to tamoxifen in estrogen receptor-positive breast cancer^83^. In other words, PHGDH inhibitors have a synergistic effect with chemotherapeutic drugs, suggesting their combination as an effective approach for treating breast cancer patients.

Adaptation is the primary obstacle in cancer metabolism. Considering this, exploiting the synergy of drug combinations in overcoming drug resistance linked to metabolism could be a novel therapeutic approach^84^. In the context of various cancer treatments and considering PHGDH expression, combining PHGDH inhibition with other therapies is one such strategy^85,86^. In thyroid cancers, the expression of proteins related to serine metabolism, including PHGDH, is more prevalent in anaplastic thyroid cancer than in papillary thyroid cancer^87^. It has been noted that anaplastic thyroid cancer does not respond effectively to monotherapy with multityrosine kinase inhibitors, and combination treatment with lenvatinib and sorafenib has been proposed as an effective approach^87,88^. In addition, a recent study suggested that coinhibition of glutamine and one-carbon metabolism further enhances the effects of lenvatinib and sorafenib^87^.

Similar to dual inhibition approaches, an extensively studied treatment strategy involves diet therapy. Combinations of inhibitors and diet therapy have exhibited therapeutic efficacy in vivo against tumors resistant to drugs or diet therapy alone by reducing one-carbon unit availability. In simpler terms, PHGDH inhibition may enhance the therapeutic effectiveness of a serine-depleted diet. In mice administered a serine- and glycine-free diet along with drug treatment, plasma serine and glycine levels were significantly decreased, and tumor growth was strongly inhibited. In addition, there was an increase in cell death. The abundance of glycine in the tumors was significantly decreased, and a consistently low abundance of serine was maintained. These findings collectively confirmed a reduction in tumor growth^11^. Targeting PHGDH alone has therapeutic effects; however, there are instances where greater efficacy is observed when PHGDH inhibition is considered in conjunction with other treatment modalities.

According to one study, S2-VP10 and PK-59 cells rapidly formed tumors in mice fed a diet without serine and glycine. Compared with mice fed a control diet, those fed a diet lacking serine and glycine showed a significant reduction in PK-59 tumor formation. The number of Ki67-positive cells in S2-VP10 tumors of mice fed a diet without serine and glycine was significantly reduced, and the number of proliferating Ki67-positive cells in S2-VP10 tumors of mice fed a diet without serine and glycine was significantly lower than that in S2-VP10 tumors of mice fed the control diet. Conversely, the number of Ki67-positive cells in PK-59 tumors was significantly lower than that in mice fed a diet without serine and glycine. Moreover, the percentage of cells expressing PHGDH in S2-VP10 tumors from mice fed a serine- and glycine-deficient diet was significantly greater than that in S2-VP10 tumors from mice fed a serine- and glycine-replete diet. Conversely, PHGDH-expressing cells were nearly absent in PK-59 tumors^60^.

Therefore, not only the use of one drug but also the combination of a previously used drug with a PHGDH inhibitor and diet therapy are effective in treating cancer through PHGDH.

However, it is imperative to acknowledge that drugs targeting PHGDH may have adverse effects in addition to their therapeutic benefits. As with any pharmacological intervention, potential risks associated with these drugs have been documented. In experimental assessments of the stability of PHGDH inhibition, mice lacking PHGDH exhibited central nervous system-related complications. Furthermore, PHGDH knock-out (KO) embryos did not exhibit an increased thickness in the dorsal horn of the spinal cord, which was accompanied by a notable reduction in immunoreactivity for βIII-tubulin, a marker of newly generated postmitotic neurons. In addition, complete inactivation of PHGDH in the spinal cord of KO embryos led to a decreased level of serine, along with concurrent decreases in γ-aminobutyric acid, glutamine, glycine, taurine, and threonine^89^. Another study revealed that mice deficient in PHGDH exhibited growth retardation and severe brain malformations, leading to embryonic lethality. Concurrently, significant defects in the morphological development of the central nervous system were observed^90^. Furthermore, the administration of NCT-503 was reported to halt embryonic growth in the mouse central nervous system model. This phenomenon was linked to the effective penetration of the blood‒brain barrier by the compound^91^. This finding demonstrates the significance of serine synthesis via the PHGDH-dependent pathway in embryonic cells, which influences cell cycle progression in diverse cell types during fetal development. In essence, this factor warrants consideration in the development of drugs targeting PHGDH.

## Conclusion

The rate-limiting enzyme PHGDH plays a crucial role in serine synthesis and exhibits significant associations with various cancers. Elevated PHGDH expression in cancer correlates with increased cancer progression, increased metastasis rates and decreased patient survival rates. Moreover, a heightened PHGDH level contributes to an increase in ROS, fostering chemoresistance. Consequently, PHGDH inhibition has demonstrated efficacy in suppressing the growth of cancer cells with PHGDH amplification or overexpression, leading to the identification of several PHGDH inhibitors.

Further research should address the unexplored structural aspects of PHGDH and improve the understanding of emerging drug resistance mechanisms. In addition, a study confirmed that the PHGDH inhibitor CBR-5884 can be used not only to treat breast cancer but also to treat epithelial ovarian cancer^92^. Therefore, additional research should be conducted to determine whether existing PHGDH inhibitors can be applied to other types of cancer in addition to those for which their efficacy is already known. This continued exploration holds promise for achieving improved outcomes in the challenging field of cancer therapeutics.
